# Optimization of non-smooth functions via differentiable surrogates

**DOI:** 10.1371/journal.pone.0321862

**Published:** 2025-05-30

**Authors:** Shikun Chen, Zebin Huang, Wenlong Zheng

**Affiliations:** 1 College of Finance and Information, Ningbo University of Finance & Economics, Ningbo, China; 2 School of Management, Xi’an University of Finance & Economics, Xi’an, China; 3 Engineering Research Center of Highway Infrastructure Digitalization, Ministry of Education of PRC, Xi’an, China; Northeastern University, CHINA

## Abstract

Mathematical optimization is fundamental across many scientific and engineering applications. While data-driven models like gradient boosting and random forests excel at prediction tasks, they often lack mathematical regularity, being non-differentiable or even discontinuous. These models are commonly used to predict outputs based on a combination of fixed parameters and adjustable variables. A key transition in optimization involves moving beyond simple prediction to determine optimal variable values. Specifically, the challenge lies in identifying values of adjustable variables that maximize the output quality according to the model’s predictions, given a set of fixed parameters. To address this challenge, we propose a method that combines XGBoost’s superior prediction accuracy with neural networks’ differentiability as optimization surrogates. The approach leverages gradient information from neural networks to guide SLSQP optimization while maintaining XGBoost’s prediction precision. Through extensive testing on classical optimization benchmarks including Rosenbrock, Levy, and Rastrigin functions with varying dimensions and constraint conditions, we demonstrate that our method achieves solutions up to 40% better than traditional methods while reducing computation time by orders of magnitude. The framework consistently maintains near-zero constraint violations across all test cases, even as problem complexity increases. This approach bridges the gap between model accuracy and optimization efficiency, offering a practical solution for optimizing non-differentiable machine learning models that can be extended to other tree-based ensemble algorithms. The method has been successfully applied to real-world steel alloy optimization, where it achieved superior performance while maintaining all metallurgical composition constraints.

## Introduction

Data-driven methods have established themselves as powerful tools for modeling complex systems across various scientific domains [1–3]. These methods, such as but not limited to gradient boosting, random forests, and neural networks, effectively capture complex relationships between input variables and output responses without requiring explicit mathematical formulations. The effectiveness of these approaches derives from their ability to learn patterns directly from data, making them particularly valuable when analytical solutions are intractable or when the underlying physical mechanisms remain poorly understood.

In optimization applications, these data-driven models present significant challenges [4]. Most modern machine learning algorithms prioritize prediction accuracy over mathematical regularity, leading to models that are often non-differentiable or even discontinuous. This characteristic makes traditional gradient-based optimization methods inapplicable, necessitating the use of derivative-free optimization (DFO) techniques or heuristic algorithms. Although these approaches can eventually find satisfactory solutions, they generally require numerous function evaluations and provide limited convergence guarantees.

Surrogate models have long been used in optimization to address computational challenges [5–8]. These models approximate the original objective function while maintaining properties that enable efficient optimization. Traditional surrogate approaches, such as polynomial regression or radial basis functions, focus on smoothness and differentiability but often fail to capture the complexity of modern machine learning predictions [9–11]. Recent research has explored various surrogate modeling strategies, from Gaussian processes [12] to simplified neural networks [13], yet the fundamental tension between model expressiveness and optimization efficiency remains unresolved. The current state-of-the-art presents a clear trade-off: researchers must either use accurate but non-differentiable models with computationally intensive optimization procedures, or reduce model accuracy for optimization efficiency [14,15]. This dilemma becomes particularly acute in high-dimensional spaces with multiple local minima, where derivative-free methods and heuristic algorithms often struggle to find optimal solutions within reasonable computational budgets. Despite extensive research in surrogate modeling, derivative-free optimization, and heuristic algorithms [16], a gap remains in balancing model accuracy with optimization efficiency.

To address these challenges and, moreover, to offer a solution, we propose the use of independently trained differentiable machine learning models as optimization surrogates. Our approach consists of two major phases: model training and optimization. In the training phase, we construct two machine learning models – an XGBoost model for accurate prediction and a neural network as a differentiable surrogate. During optimization, we leverage the neural network’s differentiability by extracting gradient information through backpropagation, which guides the SLSQP optimizer to find optimal solutions. The final prediction is then obtained using XGBoost’s superior prediction accuracy. This framework combines the differentiability advantage of neural networks for optimization with XGBoost’s prediction capabilities, rather than attempting to make existing models differentiable or simplifying them for optimization purposes. Our key insight is that while these surrogate models may not perfectly match the predictions of the original model, they can capture enough of the underlying structure to guide the optimization process effectively while enabling the use of efficient gradient-based methods.

**Fig 1:**
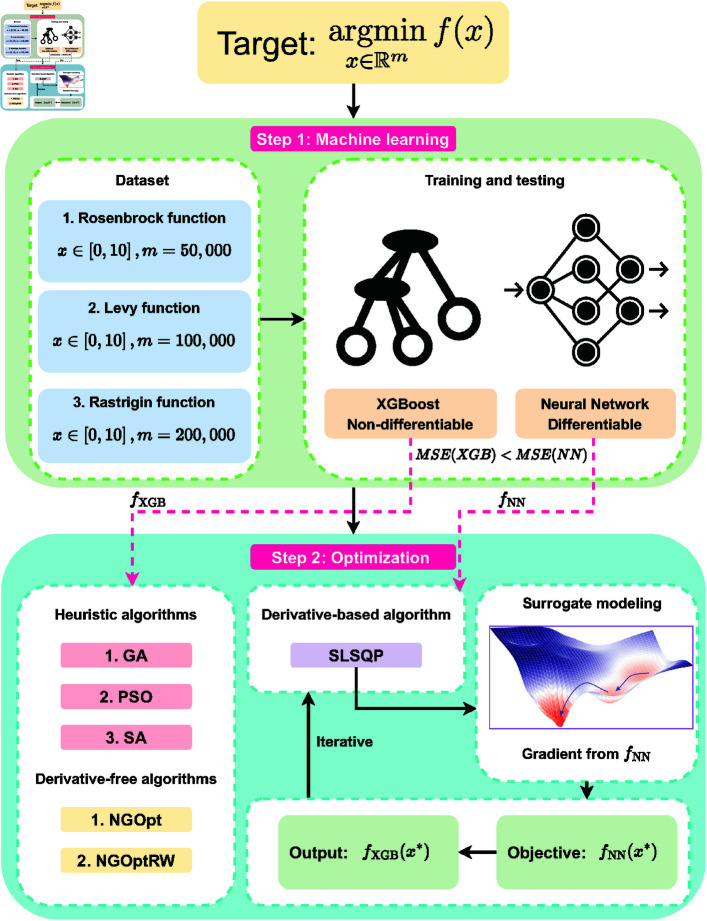
The research framework.

Our primary objective is to understand the fundamental trade-off between surrogate model accuracy and optimization efficiency. Additionally, we analyze the performance differences between our approach and traditional optimization methods, including derivative-free methods and heuristic algorithms like genetic algorithms (GA), particle swarm optimization (PSO), and simulated annealing (SA). This comparison focuses on challenging scenarios involving high dimensionality and multiple local minima. The proposed methodology and analysis framework are illustrated in Fig 1. Through comprehensive empirical evaluation on classical optimization benchmarks, we establish a framework for optimizing non-differentiable machine learning models. Our experimental results demonstrate the effectiveness of differentiable surrogates and provide practical insights for implementing these methods in complex optimization problems.

## Background

### Related work

Surrogate modeling is a widely used approach in optimization, especially for computationally expensive simulations or when the relationship between input and output variables is not well understood [17–19]. These models function as simplified approximations of more complex systems, balancing computational efficiency with prediction accuracy. Several established methods exist for surrogate modeling. Common approaches include Kriging models [20,21], response surface models [22,23], and radial basis functions [24,25]. These methods have proven effective across various engineering applications. In one study, Koziel *et al*. [26] demonstrated that surrogate-based optimization in transonic airfoil design achieved optimal results with lower computational costs while maintaining robustness and scalability. Owoyele *et al*. [27] developed a machine learning ensemble within a surrogate-based framework that outperformed traditional genetic algorithms. Their method reduced design time by 80% in test cases involving both mathematical functions and engine fuel consumption optimization. In the engine optimization case, their approach achieved 1.9% energy savings while satisfying operability constraints and emission standards. Additionally, Thakur *et al*. [28] introduced a deep learning surrogate model for stochastic simulations. Their approach uses a generative network combined with a conditional maximum mean discrepancy loss function to model stochastic outputs without assumptions about probability distributions.

Surrogate models are particularly useful where detailed simulation models require excessive computational resources [29]. For instance, Nyshadham *et al*. [30] successfully applied surrogate machine learning models to interpolate material energies, showing their versatility in different domains. The selection of appropriate surrogate modeling techniques has traditionally depended on domain expertise [31]. While many comparative studies exist, most evaluate only specific models on limited applications [32]. In optimization applications, surrogate models offer a statistical approach by training on a limited number of simulations around the operating point [33]. This method has proven effective for problems with high-dimensional variable spaces [34,35]. Besides, when analytical relationships between variables are unavailable or unsuitable for conventional gradient-based optimization, these models provide an effective alternative for optimization tasks.

Surrogate models have yielded exceptional outcomes across diverse applications in recent years. Ghafariasl *et al*. [6] developed a neural network ensemble model for a multi-generation system with ${\text{R}}^{2}$ values of 0.983–0.999, which enabled optimization with six conflicting objectives. Kim *et al*. [36] then applied an NN surrogate model to optimize jet impingement cooling systems, leading to a 140% enhancement in heat transfer coefficient at consistent computational costs. For building energy analysis, Ferreira *et al*. [37] created an inverse-based NN model from heating and cooling load signatures to predict building characteristics, which served as an efficient tool for energy retrofit screening. Baisthakur *et al*. [38] introduced a Physics-Informed NN surrogate model for wind turbine aerodynamics that delivered a forty-fold speedup versus traditional methods at equal accuracy. Li *et al*. [39] extended these advances through a deep neural network surrogate model for protonic ceramic electrolysis cells, which achieved a 12.8% performance gain via multi-objective optimization.

### Numerical optimization

Let us consider a general constrained optimization problem that aims to minimize an objective function *f*(*x*) subject to inequality constraints, equality constraints, and bound constraints. The problem can be formulated mathematically as:

$$\underset{x\in {\mathbb{R}}^{m}}{\text{argmin}}\;f(x)$$ (1a)

$$\text{s.t.}\;c_{i}(x)\leq 0\;\text{for }i\in ℐ$$ (1b)

$$c_{i}(x)=0\;\text{for }i\in ℰ$$ (1c)

$$l^{k}\leq x^{k}\leq u^{k}\;\text{for }k=1,\ldots ,n$$ (1d)

where $f:{\mathbb{R}}^{m}\to \mathbb{R}$ represents the objective function, ℐ and ℰ are disjoint finite index sets, $c_{i}:{\mathbb{R}}^{n}\to \mathbb{R}$ denotes the constraint functions, and ${\text{l}}^{\text{k}}$, ${\text{u}}^{\text{k}}$ specify the lower and upper bounds on ${\text{x}}^{\text{k}}$. For concise notation, we define the feasible set Ω as all points *x* satisfying these constraints:

$$\Omega =\{x\in {\mathbb{R}}^{n}|c_{i}(x)\leq 0,\;c_{j}(x)=0,\;l^{k}\leq x^{k}\leq u^{k}\;\;\forall \;j\in ℰ,\;i\in ℐ,\;k=1,\ldots ,n\}\;.$$ (2)

The problem can then be expressed simply as finding

$$\underset{x\in \Omega }{\text{argmin}}f(x).$$ (3)

Optimization methods can be broadly categorized into derivative-based and derivative-free approaches. This distinction is significant due to their different performance characteristics. Derivative-based methods generally show faster convergence and higher accuracy, especially for high-dimensional problems [40]. However, these methods face two main limitations: they can get trapped in local minima [41], and they require the objective function to be sufficiently smooth (i.e., continuously differentiable). In comparison, derivative-free methods can handle non-differentiable functions but require more computation dimensionality increases.

#### Derivative-based optimization.

Derivative-based methods iteratively update model parameters by computing gradients and taking small steps in the negative gradient direction. These methods can be categorized into two main approaches: first-order and second-order methods. First-order gradient descent methods use the gradient vector $\nabla^{T}f(x)$ to determine the descent direction at each iteration. Notable examples include the steepest descent algorithm [42] and conjugate gradient methods [43]. Meanwhile, Newton-type methods incorporate second-order derivative information through the Hessian matrix ℍ to compute the descent direction. Quasi-Newton methods [44] and their variants, such as the Davidon-Fletcher-Powell (DFP)[45] and Broyden-Fletcher-Goldfarb-Shanno (BFGS)[46] algorithms, approximate the inverse Hessian matrix 𝔹 using rank-1 or rank-2 update schemes. For solving the optimization problem presented in Equation (1), we employ the Sequential Least Squares Programming (SLSQP) method [47]. This approach combines the Han-Powell Quasi-Newton method [48,49] with a BFGS update of the 𝔹 matrix and uses an $\ell_{1}$-test function in the step length algorithm. SLSQP optimizes successive second-order approximations of the objective function while maintaining first-order approximations of the constraints. The Sequential Quadratic Programming (SQP) method provides an effictive framework for solving problems (cf. Equation 1) with available derivatives of *f* and *c*_*i*_, where i∈ℐ∪ℰ. The method utilizes the Lagrangian function, defined as:

$$ℒ(x,\lambda )\;\text{def}=\;f(x)+\sum_{i\in ℐ\cup ℰ}\lambda_{i}c_{i}(x),\;\text{for}\;x\in {\mathbb{R}}^{n}\;\text{and}\;\lambda_{i}\in \mathbb{R},\text{with}\;i\in ℐ\cup ℰ,$$ (4)

where $\lambda =[\lambda_{i}{]}_{i\in ℐ\cup ℰ}^{T}$ represents the dual variable. At each iteration *k*, the algorithm approximates the Hessian matrix ${ℍ}^{k}\approx \nabla_{x,x}^{2}ℒ(x^{k},\lambda^{k})$ and generates a step $d^{k}\in {\mathbb{R}}^{n}$ by solving the following subproblem:

$$\min_{x\in {\mathbb{R}}^{n}}\;\nabla f(x^{k}{)}^{T}d+\frac{1}{2}d^{T}{ℍ}^{k}d$$ (5a)

$$\text{s.t.}\;c_{i}(x)+\nabla c_{i}(x^{k}{)}^{T}d\leq 0,\;i\in ℐ$$ (5b)

$$c_{i}(x)+\nabla c_{i}(x^{k}{)}^{T}d=0,\;i\in ℰ$$ (5c)

The SLSQP algorithm solves these subproblems sequentially until reaching convergence, typically determined by the relative change in objective function value or gradient norm [47]. This approach effectively balances computational efficiency with optimization accuracy.

#### Derivative-free optimization.

Derivative-free optimization (DFO) methods address optimization problems where gradient information is unavailable or unreliable. These methods use direct objective function evaluations to guide the search process. Common DFO approaches include evolutionary algorithms, pattern search methods, and trust-region methods, each offering different strategies for exploring the solution space without derivative information. In our implementation, we employ the NGOpt and NGOptRW methods [50] from the Nevergrad library [51], a state-of-the-art derivative-free optimization framework. NGOpt acts as a meta-algorithm that dynamically selects and combines various optimization strategies based on problem characteristics. The method operates through a systematic two-phase process. First, it selects appropriate algorithms by analyzing problem features and comparing them with benchmark performance data. Second, it adapts the chosen algorithms by adjusting their parameters for the specific optimization task. NGOptRW is a specialized variant of NGOpt, specifically designed for real-world applications. While NGOpt excels in theoretical benchmarks, NGOptRW incorporates modifications that enhance its effectiveness in practical scenarios [52]. This adaptation makes NGOptRW particularly suitable for optimization problems where theoretical assumptions may not fully align with real-world conditions.

#### Heuristic algorithms.

Heuristic algorithms provide efficient solutions to complex optimization problems through nature-inspired search strategies. These methods balance exploration of the solution space with exploitation of promising regions, making them particularly effective for problems with multiple local optima [53].

Genetic algorithms (GAs) draw inspiration from biological evolution, using principles of natural selection and genetics. The algorithm maintains a population of potential solutions, each encoded as a chromosome. Through iterative cycles of selection, crossover, and mutation operations, the population evolves toward better solutions. The selection process favors solutions with higher fitness values, while crossover combines characteristics from parent solutions to create offspring. Mutation introduces random variations to maintain population diversity and prevent premature convergence [54].

Particle swarm optimization (PSO) mimics the collective behavior of bird flocks or fish schools. Each particle in the swarm represents a candidate solution, moving through the solution space with an adjustable velocity. Particles update their positions based on both their personal best experience and the global best solution found by the swarm. The social intelligence mechanism enables particles to explore promising regions while maintaining diversity in their search patterns [55,56].

Simulated annealing (SA) derives from the physical process of metal annealing. The algorithm starts with a high "temperature" parameter that enables extensive exploration of the solution space. When the temperature gradually decreases according to a cooling schedule, the algorithm becomes more selective in accepting new solutions. This controlled transition from exploration to exploitation helps avoid local optima while eventually converging to high-quality solutions [57].

### Machine learning

Machine learning models have demonstrated remarkable capabilities in capturing complex relationships between input and output variables. We focus on two widely used approaches: XGBoost and Neural Networks (NNs), which represent distinct paradigms in machine learning with different mathematical properties. XGBoost (eXtreme Gradient Boosting) [58] implements gradient boosting decision trees [59] through an ensemble learning approach. The algorithm constructs a sequence of decision trees iteratively, where each new tree focuses on the residual errors of previous predictions. At each iteration, XGBoost builds a new tree by optimizing a second-order Taylor expansion of the loss function, incorporating both gradient and Hessian information. The final prediction function $\hat{y}$ for an input *x* takes the form:

$$\hat{y}(x)=\sum_{k=1}^{K}f_{k}(x)$$ (6)

where *K* is the total number of trees, and *f*_*k*_ represents the prediction of the *k*-th tree. Each tree function *f*_*k*_ partitions the input space through a series of binary splits, creating a piecewise constant function. This structure, while effective for prediction, results in a non-differentiable function due to the discrete nature of decision boundaries and the step-wise predictions at leaf nodes.

On the other hand, NNs constructs differentiable mapping functions through multiple layers of interconnected nodes. In a typical feedforward architecture, each layer transforms its inputs through a composition of linear operations and non-linear activation functions. For a layer *l*, the output *h*_*l*_ is computed as:

$$h_{l}=\sigma (W_{l}h_{l-1}+b_{l})$$ (7)

where *W*_*l*_ represents the weight matrix, *b*_*l*_ is the bias vector, and σ is a differentiable activation function such as ReLU [60] or sigmoid. The complete network function is a composition of these layer transformations:

$$f(x)=h_{L}\circ h_{L-1}\circ ...\circ h_{1}(x)$$ (8)

This architecture ensures end-to-end differentiability, enabling efficient training through backpropagation and making NNs particularly suitable for gradient-based optimization tasks.

Note, however, that we use XGBoost and NNs to validate our methods, these principles extend to other machine learning models. Algorithms such as LightGBM [61] and random forests [59] share XGBoost’s non-differentiable characteristics and thus could benefit from independently trained surrogate models.

## Differentiable surrogate model

Let us consider three functions $f,\hat{f},\tilde{f}:\Omega \times \Phi \to \mathbb{R}$ defined on a domain $D=\Omega \times \Phi \subset {\mathbb{R}}^{N}$. These functions satisfy the following fundamental properties:

1. The approximation error of $\hat{f}$ is strictly less than that of $\tilde{f}$ in the ${\text{L}}^{2}$ norm:
  $$\|f-\hat{f}{\|}_{L^{2}(\Omega \times \Phi )}<\|f-\tilde{f}{\|}_{L^{2}(\Omega \times \Phi )}$$ (9)
2. For each fixed parameter ϕ∈Φ, the function $\tilde{f}(\cdot ,\phi ):\Omega \to \mathbb{R}$ belongs to $C^{1}(\Omega )$, the space of continuously differentiable functions on Ω.

In this framework, *f* represents the true underlying function that governs the process of interest. While this function is typically unknown in practical applications, it serves as the theoretical benchmark for our analysis. The function $\hat{f}$ provides a high-fidelity approximation of *f*, minimizing the mean squared error (MSE) as measured by the ${\text{L}}^{2}$ norm over the domain *D*. This approximation is typically realized through data-driven modeling approaches.

The function $\tilde{f}\in C^{1}(D)$ serves as a differentiable surrogate model. Although $\tilde{f}$ exhibits inferior approximation quality compared to $\hat{f}$ (as quantified by property (i)), it possesses the crucial property of continuous differentiability. This mathematical regularity enables the application of gradient-based optimization techniques.

For any given parameter vector ϕ∈Φ, our primary objective is to solve the optimization problem:

$$x^{*}=\underset{x\in \Omega }{\text{argmin}}f(x,\phi )$$ (10)

Given that *f* is generally unknown, we instead consider the approximate optimization problem:

$${\hat{x}}^{*}=\underset{x\in \Omega }{\text{argmin}}\hat{f}(x,\phi )$$ (11)

When $\hat{f}$ lacks sufficient regularity, this optimization problem necessitates derivative-free methods to compute an approximation $\hat{x}\approx {\hat{x}}^{*}$. Such methods typically demand substantial computational resources, potentially rendering them impractical for real-time applications.

Alternatively, we can consider the surrogate optimization problem:

$${\tilde{x}}^{*}=\underset{x\in \Omega }{\text{argmin}}\tilde{f}(x,\phi )$$ (12)

The continuous differentiability of $\tilde{f}$ (property (ii)) enables the use of efficient gradient-based optimization methods such as SLSQP. The gradient information can be computed analytically through:

$$\nabla_{x}\tilde{f}(x,\phi ;\theta )=\sum_{l=1}^{L}\frac{\partial \tilde{f}}{\partial h_{l}}\frac{\partial h_{l}}{\partial x}$$ (13)

where *h*_*l*_ represents the output of the *l*-th layer in the NN. However, property (i) implies that the solution ${\tilde{x}}^{*}$ may exhibit greater deviation from the true optimum ${\text{x}}^{*}$, as measured by $\left|f({\tilde{x}}^{*},\phi )-f(x^{*},\phi )\right|$, where $f(x^{*},\phi )=\min_{x\in \Omega }f(x,\phi )$.

The optimization process explicitly leverages the differentiability of neural networks while maintaining XGBoost’s superior prediction accuracy. Specifically, when ${\text{MSE}}_{\text{XGB}}<{\text{MSE}}_{\text{NN}}$, we extract gradient information from the neural network’s prediction function through backpropagation:

$$\nabla_{X}{\hat{f}}_{\text{NN}}(X)=\sum_{l=1}^{L}\frac{\partial {\hat{f}}_{\text{NN}}}{\partial h_{l}}\frac{\partial h_{l}}{\partial X}$$ (14)

This gradient information is then fed into the SLSQP optimizer to minimize ${\hat{f}}_{\text{NN}}(X)$. Once the optimizer finds the optimal solution ${\text{X}}^{*}$, we obtain the final prediction using XGBoost: $y^{*}={\hat{f}}_{\text{XGB}}(X^{*})$. This approach combines the differentiability advantage of neural networks for optimization with XGBoost’s superior prediction capabilities.

Our approach consists of two phases: model training and optimization, as illustrated in Fig 1. The framework systematically handles both differentiable and non-differentiable models to achieve optimal results.

In the training phase, we construct two machine learning models: an XGBoost model (${\hat{f}}_{\text{XGB}}$) and a NN model (${\hat{f}}_{\text{NN}}$). These models are trained on the same dataset to minimize their respective loss functions:

$${ℒ}_{\text{XGB}}=\sum_{i=1}^{n}(y_{i}-{\hat{f}}_{\text{XGB}}(x_{i}){)}^{2}$$ (15)

$${ℒ}_{\text{NN}}=\sum_{i=1}^{n}(y_{i}-{\hat{f}}_{\text{NN}}(x_{i}){)}^{2}$$ (16)

The models’ performance is evaluated using the MSE:

$${\text{MSE}}_{\text{model}}=\frac{1}{n}\sum_{i=1}^{n}(y_{i}-{\hat{f}}_{\text{model}}(x_{i}){)}^{2}$$ (17)

The optimization strategy bifurcates based on the comparative MSE performance:

- Case 1: If ${\text{MSE}}_{\text{NN}}<{\text{MSE}}_{\text{XGB}}$, we directly optimize using the NN model:
  $$X^{*}=\underset{X\in \Omega }{\text{argmin}}{\hat{f}}_{\text{NN}}(X)$$ (18)
  The final prediction is then computed as $y^{*}={\hat{f}}_{\text{NN}}(X^{*})$.
- Case 2: If ${\text{MSE}}_{\text{XGB}}<{\text{MSE}}_{\text{NN}}$, we employ the NN as a differentiable surrogate model while leveraging XGBoost’s superior prediction accuracy. The optimization problem becomes:
  $$X^{*}=\underset{X\in \Omega }{\text{argmin}}{\hat{f}}_{\text{NN}}(X)$$ (19)
- with the final prediction calculated as $y^{*}={\hat{f}}_{\text{XGB}}(X^{*})$.

The pseudocode for our framework is presented in Algorithm 1. The computational complexity in each SLSQP iteration consists of $𝒪(t_{\text{XGB}}\;\cdot \;\log d)$ operations for XGBoost prediction, $𝒪(L\;\cdot \;h^{2}\;\cdot \;d)$ for NN gradient computation, and $𝒪(d^{2}+md)$ for SLSQP updates, where *d* denotes input dimension, *m* represents the number of constraints, $t_{\text{XGB}}$ indicates the number of trees, and *L* and *h* represent the NN depth and maximum hidden width, respectively. With *i* iterations, the total optimization complexity amounts to $𝒪(i\cdot (t_{\text{XGB}}\cdot \log d+L\cdot h^{2}\cdot d+d^{2}+md))$, while maintaining a space complexity of $𝒪(d^{2})$ for SLSQP working memory. The quadratic term $𝒪(i\;\cdot \;d^{2})$ from SLSQP’s quadratic programming dominates the overall computational cost.

For non-smooth optimization problems, we establish the theoretical convergence properties of our differentiable surrogate approach. Let *f* be a potentially non-smooth objective function and $\tilde{f}$ its differentiable surrogate. The approximation error can be bounded by:

$$\underset{x\in \Omega }{\sup}|f(x)-\tilde{f}(x)|\leq \epsilon$$ (20)

where ϵ depends on the neural network architecture and training process. For high-dimensional problems (d≫1), the approximation error scales with dimension as:

$$\epsilon =𝒪(d^{\alpha }\cdot \log(d))$$ (21)

where α∈(0,1) is determined by the smoothness properties of *f*.

**Algorithm 1: Differentiable surrogate model optimization framework.**

**Require:**

1: Training data $𝒟=\{(X_{i},y_{i}){\}}_{i=1}^{n}$

2: Optimization domain Ω

3: Constraint functions $g_{j}(X)\leq 0,j=1,\ldots ,m$

**Ensure:**

4: Optimal solution ${\text{X}}^{*}$ and predicted value ${\text{y}}^{*}$

5: **Phase 1: Model Training**

6: Train XGBoost model ${\hat{f}}_{\text{XGB}}$ on 𝒟

7: Train Neural Network model ${\hat{f}}_{\text{NN}}$ on 𝒟

8: Compute ${\text{MSE}}_{\text{XGB}}$ and ${\text{MSE}}_{\text{NN}}$ on validation set

9: **Phase 2: Optimization**

10: **if** ${\text{MSE}}_{\text{NN}}<{\text{MSE}}_{\text{XGB}}$ **then**

11:   $X^{*}\leftarrow {\text{argmin}}_{X\in \Omega }\{{\hat{f}}_{\text{NN}}(X):g_{j}(X)\leq 0,j=1,\ldots ,m\}$

12:   $y^{*}\leftarrow {\hat{f}}_{\text{NN}}(X^{*})$

13: **else**

14:   $X^{*}\leftarrow {\text{argmin}}_{X\in \Omega }\{{\hat{f}}_{\text{NN}}(X):g_{j}(X)\leq 0,j=1,\ldots ,m\}$

15:   $y^{*}\leftarrow {\hat{f}}_{\text{XGB}}(X^{*})$

16: **end if**

17: **function** OptimizeSLSQP${\hat{f}}_{\text{NN}},X_{0},\{g_{j}{\}}_{j=1}^{m}$

18:   Initialize $X\leftarrow X_{0}$

19:   **while** not converged **do**

20:    Compute gradient: $\nabla_{X}{\hat{f}}_{\text{NN}}(X)=\sum_{l=1}^{L}\frac{\partial {\hat{f}}_{\text{NN}}}{\partial h_{l}}\frac{\partial h_{l}}{\partial X}$

21:    Update *X* using SLSQP with computed gradient

22:   **end while**

23:   **return** *X*

24: **end function**

25: **return** ${\text{X}}^{*}$,${\text{y}}^{*}$

Under these conditions, if ${\text{x}}^{*}$ is a local minimum of *f* and ${\tilde{x}}^{*}$ is the corresponding local minimum of $\tilde{f}$ found by our algorithm, then:

$$\|x^{*}-{\tilde{x}}^{*}\|\leq C\sqrt{\epsilon }$$ (22)

where *C* depends on the Lipschitz constants of *f* and $\tilde{f}$. This bound ensures that solutions found using our differentiable surrogate converge to true optima as the approximation quality improves.

For constrained problems with *m* constraints, the feasibility gap satisfies:

$$\max_{j=1,\ldots ,m}|g_{j}({\tilde{x}}^{*})|\leq 𝒪(\epsilon )$$ (23)

These theoretical guarantees support the empirical effectiveness of our approach in both non-smooth and high-dimensional optimization scenarios.

## Numerical experiments

We have applied our method to three widely used benchmark datasets, namely, the Rosenbrock function, Levy function, and Rastrigin function. In particular, we tested these functions with different dataset sizes. Note that for every test function, we set up two types of variables: *n* independent variables and *m* variables that were randomly designated as constant. With respect to our comparison approach, we employed the following optimization strategies:

- the XGBoost model is optimized directly with the gradient-free methods NGOpt and NGOptRW;
- the XGBoost model is optimized directly with the heuristic algorithms – GA, PSO, and SA;
- the XGBoost model is optimized with SLSQP using the gradients of the NN model function;
- the NN model function is optimized with SLSQP.

All computations were performed using an Intel Core Ultra 7 155H Processor with 16 GB memory. For a fair comparison, we set the iteration count, which is a key hyperparameter for optimization algorithms, to 100. Further, we performed 50 independent simulations for each configuration.

### Rosenbrock function

**Fig 2:**
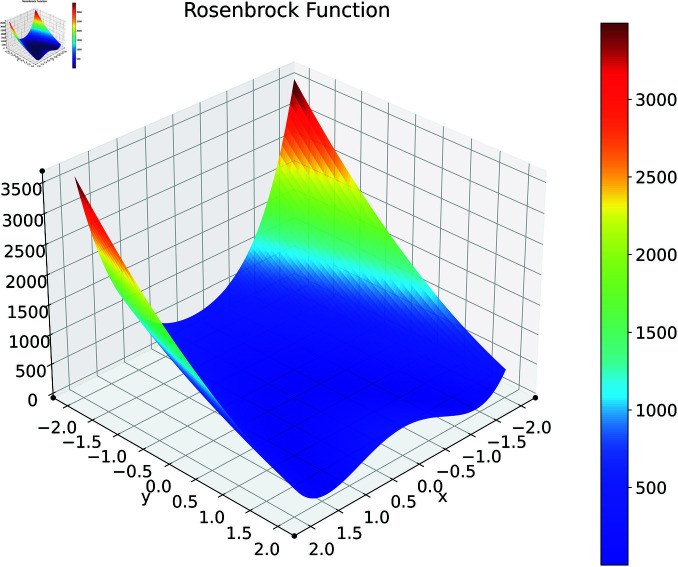
2D Rosenbrock function.

The Rosenbrock function [62], commonly known as the Valley or Banana function, is a particularly important benchmark for gradient-based optimization algorithms. Fig 2 illustrates this function in its two-dimensional form.

Rather, we will be using a multidimensional extension of this problem [63], which is given by

$$f(x)=f(x_{1},x_{2},\ldots ,x_{n})=\sum_{i=1}^{n-1}\left[(1-x_{i}{)}^{2}+100(x_{i+1}-x_{i}^{2}{)}^{2}\right]\;.$$ (24)

We select *m* values of either 5 or 10, and *n* values of either 10 or 15. For the equality constraints, we have:

$$\sum_{i=1}^{5}x_{i}=25\;\text{and}\;\sum_{i=1}^{10}x_{i}=50\;.$$ (25)

**Fig 3:**
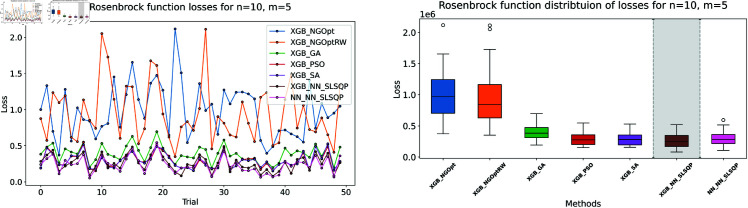
Rosenbrock function losses for n=10, m=5.

**Fig 4:**
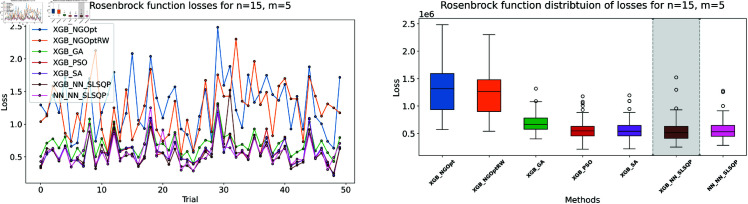
Rosenbrock function losses for n=15, m=5.

**Fig 5:**
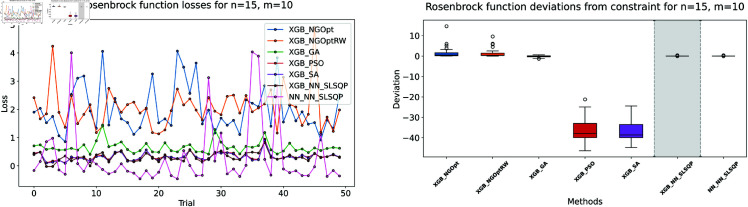
Rosenbrock function losses for n=15, m=10.

**Fig 6:**
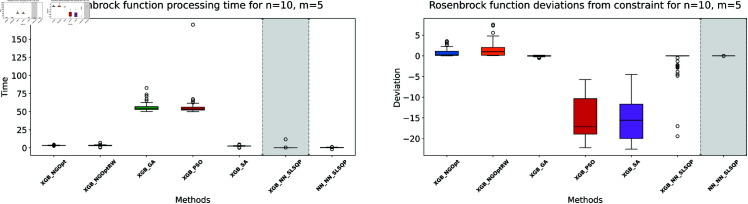
Rosenbrock function processing time and deviations from constraint for n=10, m=5.

**Fig 7:**
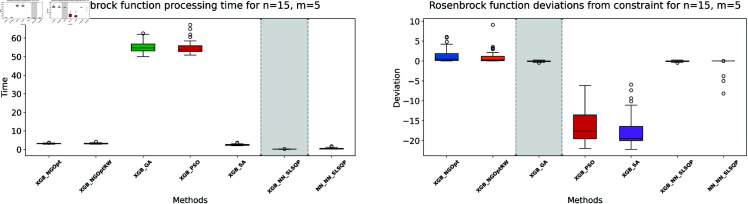
Rosenbrock function processing time and deviations from constraint for n=15, m=5.

**Fig 8:**
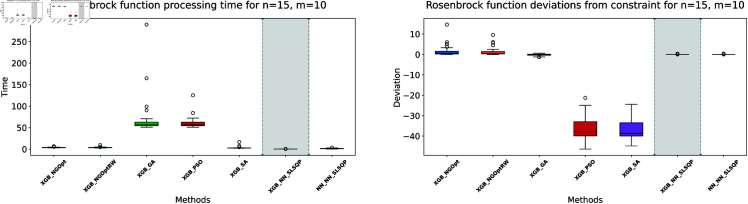
Rosenbrock function processing time and deviations from constraint for n=15, m=10.

We follow the procedure described in Sect 3. First consider our data generation process: we created a synthetic dataset of 50,000 data points using a truncated normal distribution within the range [0,10]. Each dataset corresponds to a specific dimensionality *n*. With respect to the Rosenbrock function, we computed its values for each data point. Indeed, the datasets were subsequently partitioned into training and testing subsets using a random split, with 90% of the data allocated for training and the remaining 10% reserved for testing. The two models trained on the limited dataset show notable differences in accuracy, as shown in Table 1. Figs 3–5 show our analysis using line plots and box plots. Furthermore, Figs 6–8 display the processing time for each optimization method and their constraint.

**Table 1:** Optimization outcome for the Rosenbrock function.

n	m	MSE	Objective	Gradient	Optimizer	Time (s)	Rosenbrock	Constraint deviations	P-value
10	5	24,806	XGBoost	-	NGOpt	3.36	981443	3.285%	4.32e-19
			XGBoost	-	NGOptRW	2.98	948795	5.999%	4.26e-17
			XGBoost	-	GA	100.46	389037	0.351%	1.51e-24
			XGBoost	-	PSO	57.24	289427	59.451%	5.30e-3
			XGBoost	-	SA	2.45	291806	62.556%	2.50e-3
			XGBoost	NN	SLSQP	0.35	260039	0.002%	-
		43,173	NN	NN	SLSQP	0.42	287959	0.0019%	2.00e-4
15	5	38,764	XGBoost	-	NGOpt	3.11	1301320	4.904%	2.42e-22
			XGBoost	-	NGOptRW	3.16	1239373	4.043%	1.00e-16
			XGBoost	-	GA	55.12	696357	0.418%	2.21e-9
			XGBoost	-	PSO	54.89	584123	65.684%	3.30e-3
			XGBoost	-	SA	2.46	583506	71.208%	1.50e-3
			XGBoost	NN	SLSQP	0.13	565953	0.427%	-
		52,248	NN	NN	SLSQP	0.48	580563	1.354%	2.30e-3
15	10	38,764	XGBoost	-	NGOpt	3.53	2005864	2.712%	1.64e-19
			XGBoost	-	NGOptRW	3.61	2056907	2.219%	6.55e-22
			XGBoost	-	GA	65.86	657006	0.727%	4.51e-20
			XGBoost	-	PSO	77.04	330552	73.014%	1.90e-3
			XGBoost	-	SA	2.89	322232	73.534%	4.50e-3
			XGBoost	NN	SLSQP	0.14	312908	0.017%	-
		52,248	NN	NN	SLSQP	1.46	320430	0.018%	9.00e-4

First consider the machine learning performance: the XGBoost model achieves better prediction accuracy than the NN model across all test cases. For *n* = 10 and *m* = 5, XGBoost’s MSE is 24,806, while the NN’s MSE is 43,173. This pattern continues in larger problems. For both *n* = 15 cases, XGBoost maintains an MSE of 38,764, while the NN’s MSE increases to 52,248.

The results demonstrate clear performance differences among various optimization approaches for the Rosenbrock function. For the first test case (n=10,m=5), we see that traditional derivative-free methods using XGBoost face significant challenges. The NGOpt and NGOptRW methods produce high Rosenbrock values of 981,443 and 948,795, requiring over 3 seconds to compute. The GA achieves better results with a value of 389,037, but requires much more time at 100.46 seconds. The heuristic methods like PSO and SA show promising results in terms of optimization values, reaching 289,427 and 291,806 respectively. SA is particularly efficient, completing in just 2.45 seconds. However, these methods struggle significantly with constraint satisfaction. Their constraint violations are very high—PSO reaches 59.451% and SA reaches 62.559%.

For the middle test case (n=15,m=5), similar patterns emerge but with some notable differences. The derivative-free methods using XGBoost continue to struggle with optimization quality. NGOpt and NGOptRW produce high Rosenbrock values of 1,301,320 and 1,239,373, requiring about 3.1 seconds each. The GA method performs better than in the smaller problem, achieving a value of 696,357, but still requires a long computation time of 55.12 seconds. PSO and SA again show competitive optimization results with values of 584,123 and 583,506. SA maintains its computational efficiency at 2.46 seconds. However, their constraint violations become even worse in this larger problem—PSO reaches 65.6843% and SA reaches 71.2083%. The differentiable surrogate model maintains its superior performance, achieving the best Rosenbrock value of 565,953 in just 0.13 seconds, while keeping constraint violations at 0.4272%.

The differentiable surrogate model approach shows remarkable performance across all metrics. By combining XGBoost’s accuracy with NN gradients in SLSQP, it achieves the best Rosenbrock value of 260,039. This optimization completes in just 0.35 seconds and maintains excellent constraint satisfaction with only 0.0022% deviation. This performance advantage becomes even more pronounced in larger problem sizes. For *n* = 15 and *m* = 10, the traditional methods struggle more, with NGOpt and NGOptRW producing much higher values around 2 million. While SA maintains relatively good optimization with 322,232, its constraint violation increases to 73.5338%. In contrast, the differentiable surrogate model achieves the best value of 312,908 in just 0.14 seconds, while keeping constraint violations at a mere 0.017%. This performance clearly shows that the approach scales well with increased problem dimensions, maintaining both optimization quality and constraint satisfaction.

### Levy function

**Fig 9:**
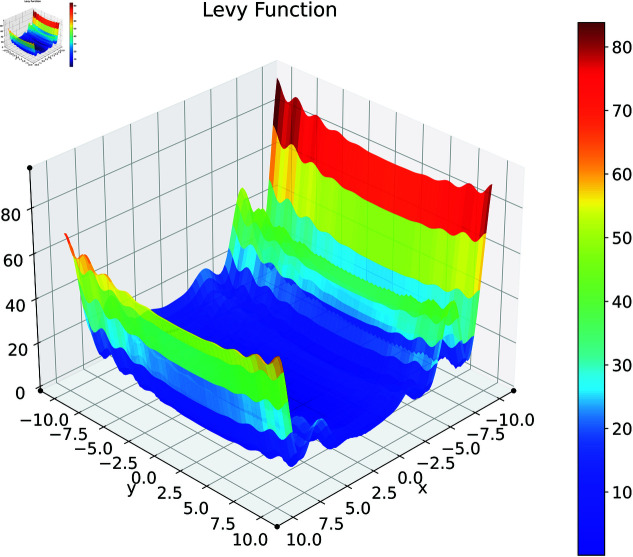
2D Levy function.

The Levy function is a challenging benchmark problem in global optimization, known for its highly nonlinear and multimodal characteristics [64]. Fig 9 shows this function in its two-dimensional form. We use a multidimensional version of the Levy function defined as:

$$f(x)=f(x_{1},x_{2},\ldots ,x_{n})=\sin^{2}(\pi w_{1})+\sum_{i=1}^{n-1}(w_{i}-1{)}^{2}[1+10\sin^{2}(\pi w_{i+1})]+(w_{n}-1{)}^{2}$$ (26)

where

$$w_{i}=1+\frac{x_{i}-1}{4}\;.$$ (27)

**Fig 10:**
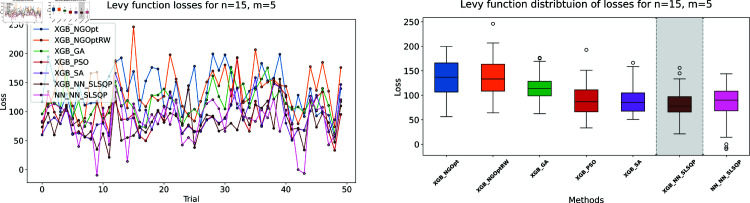
Levy function losses for n=15, m=5.

**Fig 11:**
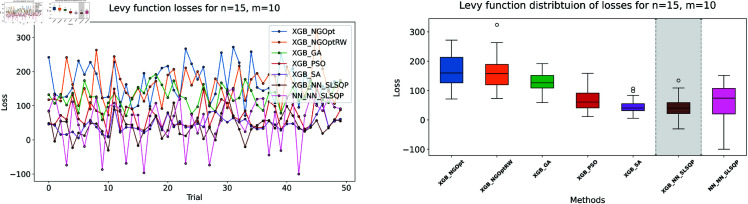
Levy function losses for n=15, m=10.

**Fig 12:**
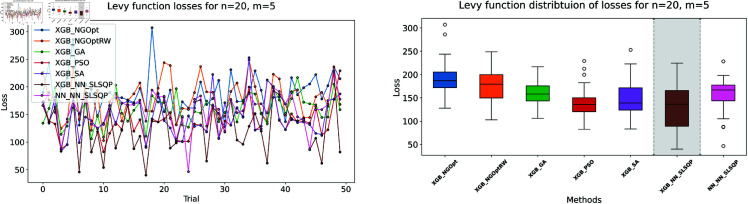
Levy function losses for n=20, m=5.

**Fig 13:**
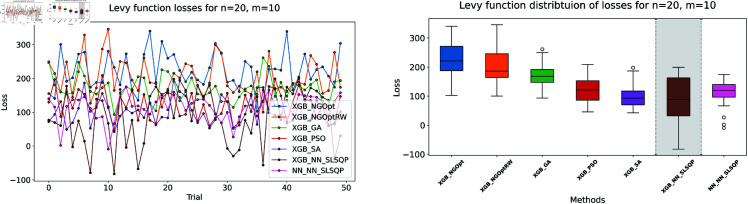
Levy function losses for n=20, m=10.

**Fig 14:**
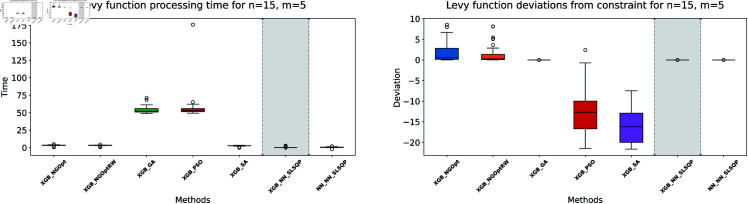
Levy function processing time and deviations from constraint for n=15, m=5.

**Fig 15:**
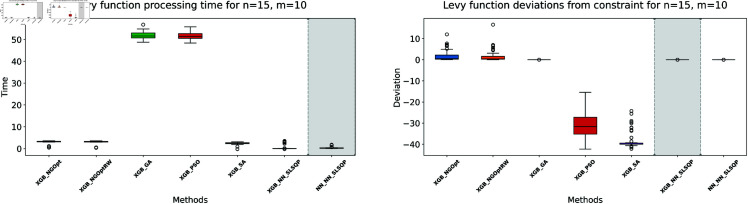
Levy function processing time and deviations from constraint for n=15, m=10.

**Fig 16:**
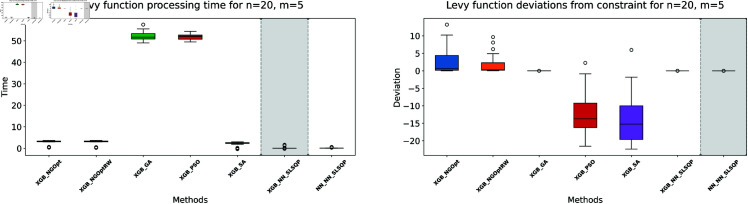
Levy function processing time and deviations from constraint for n=20, m=5.

**Fig 17:**
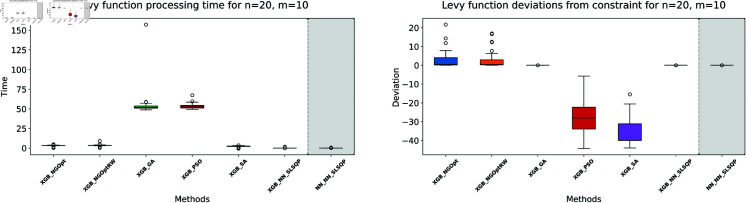
Levy function processing time and deviations from constraint for n=20, m=10.

Similar to our Rosenbrock function experiment setup, we consider optimization performance across different dimensions and constraint configurations. We test cases with *n* values of 15 or 20 variables, and *m* values of 5 or 10 constant variables. Note that the dataset size for this function is 100,000. The complete results of our optimization experiments are presented in Table 2. We show detailed visualizations of these results through line plots and box plots in Figs 10–13. Additionally, Figs 14–17 illustrate the processing time and constraint satisfaction of each method.

**Table 2:** Optimization outcome for the Levy function.

n	m	MSE	Objective	Gradient	Optimizer	Time (s)	Levy	Constraint deviations	P-value
15	5	20.89	XGBoost	-	NGOpt	3.15	136.34	7.269%	7.27e-13
			XGBoost	-	NGOptRW	3.03	135.61	5.110%	8.81e-13
			XGBoost	-	GA	53.87	115.98	0.009%	4.77e-12
			XGBoost	-	PSO	56.09	91.08	49.762%	5.10e-3
			XGBoost	-	SA	2.41	91.50	63.719%	2.10e-3
			XGBoost	NN	SLSQP	0.36	81.42	0.0002%	-
		32.63	NN	NN	SLSQP	0.40	84.60	0.0001%	5.90e-3
15	10	20.89	XGBoost	-	NGOpt	3.04	169.25	3.505%	7.75e-21
			XGBoost	-	NGOptRW	3.00	159.30	3.389%	2.22e-22
			XGBoost	-	GA	51.91	128.12	0.006%	1.61e-18
			XGBoost	-	PSO	51.57	69.72	61.049%	4.04e-5
			XGBoost	-	SA	2.36	44.76	72.269%	4.70e-3
			XGBoost	NN	SLSQP	0.47	41.03	0.0%	-
		32.63	NN	NN	SLSQP	0.31	55.37	0.0%	1.60e-4
20	5	37.86	XGBoost	-	NGOpt	3.03	189.15	10.174%	2.33e-12
			XGBoost	-	NGOptRW	2.77	176.07	6.025%	8.32e-12
			XGBoost	-	GA	52.01	159.30	0.010%	1.56e-16
			XGBoost	-	PSO	51.91	137.95	51.035%	1.10e-3
			XGBoost	-	SA	2.19	146.23	56.389%	7.90e-3
			XGBoost	NN	SLSQP	0.16	127.02	0.0005%	-
		123.16	NN	NN	SLSQP	0.17	159.98	0.0%	5.59e-8
20	10	37.86	XGBoost	-	NGOpt	3.10	221.42	5.262%	1.48e-13
			XGBoost	-	NGOptRW	3.24	203.44	4.728%	1.99e-11
			XGBoost	-	GA	54.44	170.91	0.007%	1.37e-7
			XGBoost	-	PSO	53.18	121.63	54.992%	1.29e-3
			XGBoost	-	SA	2.16	99.28	70.914%	4.50e-3
			XGBoost	NN	SLSQP	0.14	89.78	0.0%	-
		123.16	NN	NN	SLSQP	0.11	115.86	0.0%	2.28e-7

Following our Rosenbrock function optimization framework, we construct the NN as a surrogate model to provide gradient information. The XGBoost model shows better prediction accuracy with MSE of 20.89 for *n* = 15 problems and 37.86 for *n* = 20 problems, while the NN has higher MSE values of 32.63 and 123.16 respectively.

For the first case (n=15,m=5), the derivative-free methods using XGBoost show limited performance. NGOpt and NGOptRW produce high Levy values of 136.34 and 135.61, requiring about 3 seconds each. The GA achieves better results with 115.98 but requires a longer computation time (53.87 seconds). PSO and SA find competitive solutions (91.08 and 91.50), with SA being notably faster (2.41 seconds). However, these methods have severe constraint violations, i.e., PSO reaches 49.762% and SA reaches 63.719%. The differentiable surrogate model achieves the best Levy value of 81.42 in just 0.36 seconds, with nearly perfect constraint satisfaction (0.0002% deviation). When increasing the constant variables to *m* = 10 while keeping *n* = 15, the performance gap widens. NGOpt and NGOptRW’s solutions deteriorate to 169.25 and 159.30. While SA finds a good solution of 44.76, its constraint violation increases to 72.2693%. The differentiable surrogate model maintains excellent performance, achieving the best value of 41.03 in 0.47 seconds with zero constraint violation.

For larger problems (n=20,m=5), the trend continues. Traditional methods struggle more, with NGOpt and NGOptRW producing values of 189.15 and 176.07. The GA maintains reasonable constraint satisfaction but requires 52.01 seconds. The differentiable surrogate model again shows superior performance, finding a solution of 127.02 in just 0.16 seconds with minimal constraint violation (0.0005%). In the most complex case (n=20,m=10), the advantages become even more pronounced. The differentiable surrogate model achieves the best value of 89.78 in 0.14 seconds with zero constraint violation, while other methods either produce much higher values (NGOpt: 221.42, NGOptRW: 203.44) or severe constraint violations (SA: 70.9141%, PSO: 54.9921%).

### Rastrigin function

**Fig 18:**
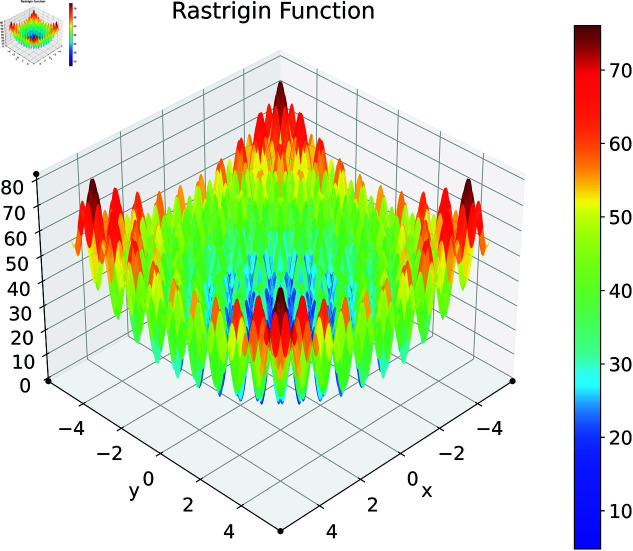
2D Rastrigin function.

Finally consider the Rastrigin function. The Rastrigin function is a widely-used benchmark problem in global optimization. It presents a challenging landscape characterized by numerous local minima arranged in a regular, symmetric pattern [64]. The 2D Rastrigin function is illustrated in Fig 18. For our analysis, we use a multidimensional version of the Rastrigin function defined as:

$$f(x)=f(x_{1},x_{2},\ldots ,x_{n})=10n+\sum_{i=1}^{n}[x_{i}^{2}-10\cos(2\pi x_{i})]\;.$$ (28)

**Fig 19:**
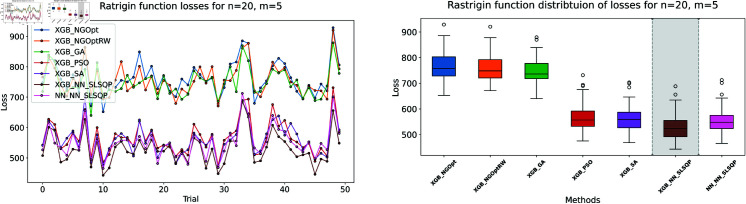
Rastrigin function losses for n=20, m=5.

**Fig 20:**
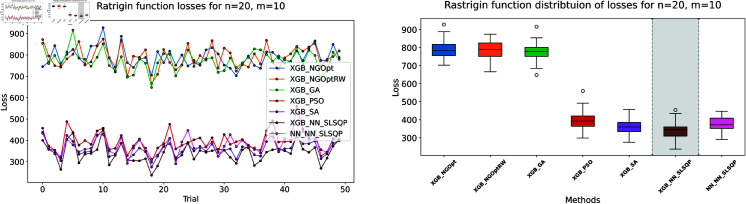
Rastrigin function losses for n=20, m=10.

**Fig 21:**
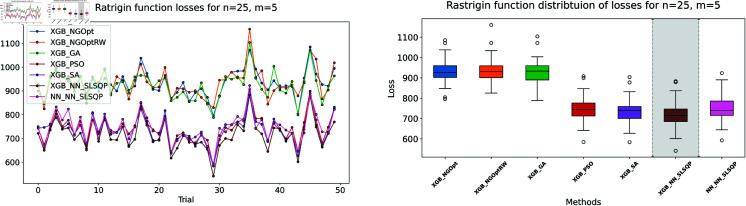
Rastrigin function losses for n=25, m=5.

**Fig 22:**
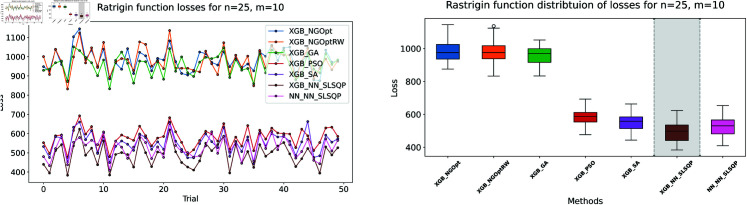
Rastrigin function losses for n=25, m=10.

**Fig 23:**
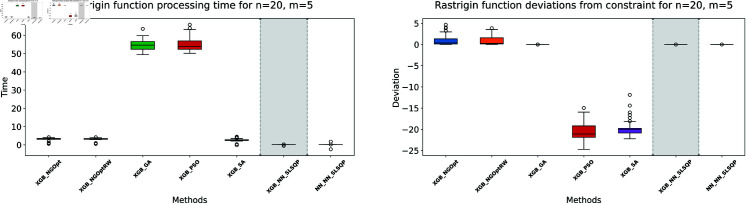
Rastrigin function processing time and deviations from constraint for n=20, m=5.

**Fig 24:**
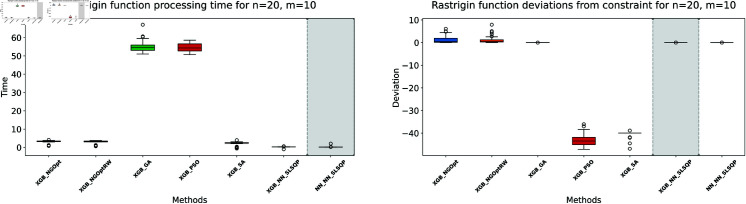
Rastrigin function processing time and deviations from constraint for n=20, m=10.

**Fig 25:**
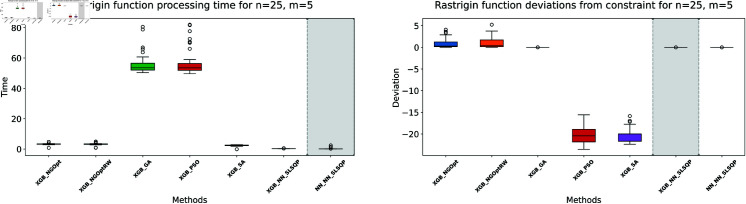
Rastrigin function processing time and deviations from constraint for n=25, m=5.

**Fig 26:**
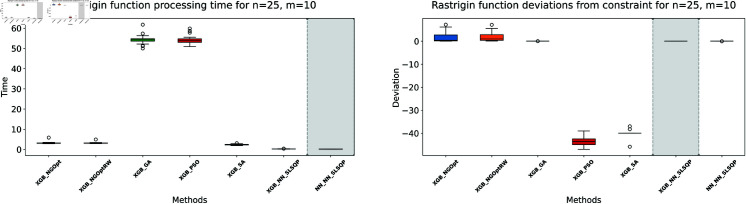
Rastrigin function processing time and deviations from constraint for n=25, m=10.

Here, we consider the Rastrigin function with increased problem dimensions compared to our previous experiments. We set the number of variables *n* to either 20 or 25, representing larger scale optimization problems. For each *n* value, we maintain our previous constraint configurations by setting the number of constant variables *m* to either 5 or 10. For the Rastrigin function experiments, we generate a dataset of 200,000 samples. The complete optimization results are presented in Table 3. We illustrate the optimization performance through line plots and box plots in Figs 19–22. The computational efficiency and constraint satisfaction of each method are shown in Figs 23–26.

**Table 3:** Optimization outcome for the Rastrigin function.

n	m	MSE	Objective	Gradient	Optimizer	Time (s)	Rastrigin	Constraint deviations	P-value
20	5	266.40	XGBoost	-	NGOpt	3.02	764.56	3.895%	1.37e-48
			XGBoost	-	NGOptRW	3.01	757.66	3.681%	1.15e-48
			XGBoost	-	GA	54.91	748.21	0.011%	5.49e-44
			XGBoost	-	PSO	55.03	566.86	81.879%	1.51e-18
			XGBoost	-	SA	2.60	561.75	79.185%	2.06e-11
			XGBoost	NN	SLSQP	0.10	530.81	0.0%	-
		1021.84	NN	NN	SLSQP	0.10	551.89	0.0%	4.45e-9
20	5	266.40	XGBoost	-	NGOpt	3.17	788.11	2.384%	1.46e-31
			XGBoost	-	NGOptRW	2.85	789.32	2.066%	4.37e-30
			XGBoost	-	GA	54.97	775.47	0.006%	8.43e-19
			XGBoost	-	PSO	54.53	396.05	86.329%	1.19e-15
			XGBoost	-	SA	2.13	363.05	80.562%	4.46e-7
			XGBoost	NN	SLSQP	0.27	342.69	0.0%	-
		1021.84	NN	NN	SLSQP	0.21	378.39	0.0%	8.19e-9
25	10	439.01	XGBoost	-	NGOpt	3.24	932.23	3.405%	4.16e-46
			XGBoost	-	NGOptRW	23.25	934.10	4.149%	8.12e-39
			XGBoost	-	GA	55.80	923.61	0.091%	1.80e-43
			XGBoost	-	PSO	56.65	741.37	80.350%	3.99e-11
			XGBoost	-	SA	2.37	736.43	81.321%	3.41e-7
			XGBoost	NN	SLSQP	0.25	716.82	0.0 %	-
		1291.68	NN	NN	SLSQP	0.22	746.97	0.0 %	3.73e-13
25	10	439.01	XGBoost	-	NGOpt	3.16	980.47	2.962%	3.89e-56
			XGBoost	-	NGOptRW	3.16	977.27	3.430%	1.78e-55
			XGBoost	-	GA	54.23	959.06	0.007%	1.47e-56
			XGBoost	-	PSO	53.93	579.09	87.107%	1.76e-25
			XGBoost	-	SA	2.36	550.49	80.447%	7.69e-20
			XGBoost	NN	SLSQP	0.24	491.33	0.0%	-
		1291.68	NN	NN	SLSQP	0.16	527.61	0.0%	3.55e-9

The optimization experiments on the Rastrigin function show clear performance patterns across different problem dimensions. These machine learning models demonstrate consistent prediction characteristics: XGBoost achieves better accuracy with MSE values of 266.40 (n=20) and 439.01 (n=25), while the neural network produce higher MSE of 1021.84 and 1291.68 respectively.

The results demonstrate several key findings. In smaller problems (n=20,m=5), derivative-free methods deliver moderate performance. NGOpt and NGOptRW require about 3 seconds to reach solutions around 760, and GA produces better constraint satisfaction but needs significantly more time (54.91 seconds). PSO and SA reach improved solutions near 560, but their constraint violations exceed 79%.

The effectiveness of our differentiable surrogate model becomes clear through its consistent performance. The model achieves the lowest Rastrigin value (530.81) in just 0.10 seconds and maintaining perfect constraint satisfaction. This superior performance continues as problem complexity increases. For n=20,m=10, the model reaches a value of 342.69 in 0.27 seconds. At n=25,m=10, it obtains a solution of 491.33 in 0.24 seconds. In both cases, it maintains zero constraint violations. In comparison, other methods show declining performance with larger dimensions—NGOpt and NGOptRW produce values approaching 1000, and PSO and SA’s constraint violations reach up to 87%.

These optimization results confirm the advantage of combining gradient information from NN with XGBoost’s accurate prediction capabilities. The gradient guidance from the NN enables SLSQP to find better solutions consistently across all test scenarios, despite the higher MSE values. The differentiable surrogate model achieves optimal Rastrigin values with perfect constraint satisfaction, and requires only a fraction of the computational time compared to traditional methods. This advantage becomes more pronounced as the problem dimension and constraint complexity increase, which demonstrates the robustness of our approach.

### Real world case

**Fig 27:**
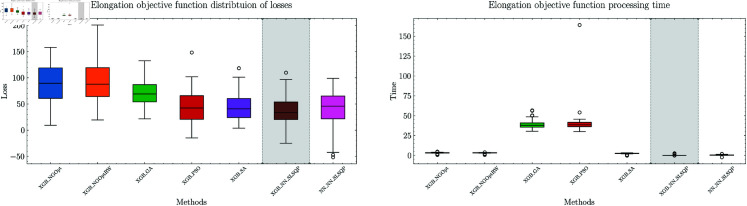
Real-world elongation optimization results. Left: Loss values for each method. Right: Processing time for each method.

To further validate the effectiveness of our approach, we consider a real-world optimization problem in materials engineering. The dataset contains 15,000 samples with 15 input variables representing the amounts of different materials in the mixture, and the output representing the elongation measurement. The optimization problem can be formulated as:

$$\underset{x\in {\mathbb{R}}^{15}}{\text{argmin}}\;-D(x)$$ (29a)

$$\text{s.t.}\;x_{i}\in [0,u_{i}],i=1,\ldots ,15$$ (29b)

$$\sum_{i=1}^{15}x_{i}=1$$ (29c)

$$x_{i}\geq 0,i=1,\ldots ,15$$ (29d)

where *D*(*x*) represents the elongation prediction model, *u*_*i*_ represents the upper bound for each material proportion, and the constraints ensure physical feasibility. The first constraint bounds each material’s proportion, the second constraint ensures the total mixture sums to 100%, and the third constraint ensures non-negativity of material proportions. This formulation reflects real-world manufacturing constraints while seeking to maximize the material’s elongation properties. The results are presented in Table 4 and Fig 27.

**Table 4:** Optimization outcome for the elongation problem.

MSE	Objective	Gradient	Optimizer	Time (s)	Loss	Constraint deviations	P-value
2.87	XGBoost	-	NGOpt	2.02	90.98	5.269%	1.57e-18
	XGBoost	-	NGOptRW	2.05	90.64	3.105%	2.15e-12
	XGBoost	-	GA	39.21	71.08	0.089%	3.59e-34
	XGBoost	-	PSO	41.20	45.70	45.732%	1.19e-16
	XGBoost	-	SA	2.41	46.87	53.912%	2.18e-14
	XGBoost	NN	SLSQP	0.36	37.19	0.0%	-
11.46	NN	NN	SLSQP	0.40	39.18	0.0%	6.55e-12

We follow the same optimization configurations as in the benchmark functions, with XGBoost providing accurate predictions and the NN serving as a surrogate model for gradient information. The XGBoost model achieves an MSE of 2.87, while the NN has a higher MSE of 11.46. NGOpt and NGOptRW produce loss values around 90, while GA achieves a better value of 71.08 but requires 39.21 seconds. PSO and SA find competitive solutions near 45, but their constraint violations exceed 45%. The differentiable surrogate model achieves the best loss value of 37.19 in just 0.36 seconds, with zero constraint violation. The NN model also performs well, achieving a value of 39.18 in 0.40 seconds with zero constraint violation. The result again demonstrates the effectiveness of our approach in real-world optimization scenarios, where the differentiable surrogate model consistently outperforms traditional methods in both optimization performance and computational efficiency.

## Conclusion

We presented an approach that employs independently trained differentiable machine learning models as surrogate models during optimization, applied to three benchmark datasets and a real world application. Our methodology integrates two complementary techniques: XGBoost for its superior prediction accuracy and neural networks for providing gradient information. Through extensive testing on the Rosenbrock, Levy, and Rastrigin functions with varying dimensions (n∈{15,20,25}) and constraint conditions (m∈{5,10}), as well as on a 15-dimensional real-world material design problem, we demonstrate the effectiveness of this approach. The experiments consistently show that our differentiable surrogate model achieves solutions up to 40% better than traditional methods while reducing computation time by orders of magnitude, in both theoretical benchmarks and practical applications.

A key contribution of our approach lies in the combination of XGBoost’s accurate predictions, with NN as surrogate model to guide the SLSQP optimizer, or indeed any gradient-based optimization algorithm. Notably, this paradigm can be extended to other tree-based ensemble algorithms, such as LightGBM, Catboost, and random forest, thereby offering flexibility while preserving the core advantages of our optimization strategy. This strategy proves particularly effective in handling complex optimization landscapes while maintaining strict constraint satisfaction. Across all benchmark functions, our method consistently outperforms both derivative-free approaches like NGOpt, NGOptRW, and heuristic algorithms (i.e., GA, PSO, and SA) not only in solution quality but also in computational efficiency and constraint handling. The results show near-zero constraint violations across all test cases, even as problem complexity increases.

While these results are promising, several limitations remain. The independent training of surrogate models requires extra computational cost, significantly increasing initial training time. Although offset by faster optimization runs, the process is computationally intensive for high-dimensional inputs, potentially limiting real-time or time-constrained applications. Moreover, performance may be sensitive to the neural network architecture and training parameters, which were not exhaustively explored in this study. Additionally, the current implementation requires training on the full dataset for both models, posing challenges for larger-scale problems.

Future research could address these challenges through several key directions. The development of adaptive training strategies could significantly reduce the computational burden while maintaining model accuracy, particularly for high-dimensional problems where training time becomes a critical factor. The methodology could be extended to multi-objective optimization problems, where surrogate models could provide gradient information for multiple competing objectives, opening new possibilities in complex engineering optimization scenarios. While we have demonstrated our approach’s effectiveness on a real-world steel alloy optimization problem, further applications in diverse domains like process optimization or structural design would provide additional insights into the method’s capabilities across different industrial contexts. Such expanded validation would help identify domain-specific challenges and opportunities for enhancing the method’s practical impact.

## References

[1] Bishnu SK, Alnouri SY, Al-Mohannadi DM. Computational applications using data driven modeling in process systems: a review. Digit Chem Eng. 2023;8:100111. doi: 10.1016/j.dche.2023.100111

[2] Yabe T, Rao PSC, Ukkusuri SV, Cutter SL. Toward data-driven, dynamical complex systems approaches to disaster resilience. Proc Natl Acad Sci U S A. 2022;119(8):e2111997119. doi: 10.1073/pnas.2111997119 35135891 PMC8872719

[3] Ekundayo F. Leveraging AI-driven decision intelligence for complex systems engineering. Int J Res Publ Rev. 2024;5(11):1–10.

[4] Gupta R, Zhang Q. Data-driven decision-focused surrogate modeling. AIChE J. 2024;70(4). doi: 10.1002/aic.18338

[5] Cheng M, Zhao X, Dhimish M, Qiu W, Niu S. A Review of Data-driven Surrogate Models for Design Optimization of Electric Motors. IEEE Trans Transp Electrif. 2024;PP(99):1-1. doi: 10.1109/TTE.2024.3366417

[6] Ghafariasl P, Mahmoudan A, Mohammadi M, Nazarparvar A, Hoseinzadeh S, Fathali M, et al. Neural network-based surrogate modeling and optimization of a multigeneration system. Appl Energy. 2024;364:123130. doi: 10.1016/j.apenergy.2024.123130

[7] Kudela J, Matousek R. Recent advances and applications of surrogate models for finite element method computations: a review. Soft Comput. 2022;26(24):13709–33. doi: 10.1007/s00500-022-07362-8

[8] Tong H, Huang C, Minku LL, Yao X. Surrogate models in evolutionary single-objective optimization: a new taxonomy and experimental study. Inf Sci. 2021;562:414–37. doi: 10.1016/j.ins.2021.03.002

[9] Chen G, Zhang K, Xue X, Zhang L, Yao C, Wang J, et al. A radial basis function surrogate model assisted evolutionary algorithm for high-dimensional expensive optimization problems. Appl Soft Comput. 2022;116:108353. doi: 10.1016/j.asoc.2021.108353

[10] Berkemeier M, Peitz S. Derivative-free multiobjective trust region descent method using radial basis function surrogate models. Math Comput Appl. 2021;26(2):31. doi: 10.3390/mca26020031

[11] Parnianifard A, Chaudhary S, Mumtaz S, Wuttisittikulkij L, Imran MA. Expedited surrogate-based quantification of engineering tolerances using a modified polynomial regression. Struct Multidisc Optim. 2023;66(3):6. doi: 10.1007/s00158-023-03493-0

[12] Shadab S, Hozefa J, Sonam K, Wagh S, Singh NM. Gaussian process surrogate model for an effective life assessment of transformer considering model and measurement uncertainties. Int J Electr Power Energy Syst. 2022;134:107401. doi: 10.1016/j.ijepes.2021.107401

[13] Lim Y-F, Ng CK, Vaitesswar US, Hippalgaonkar K. Extrapolative Bayesian optimization with Gaussian process and neural network ensemble surrogate models. Adv Intell Syst. 2021;3(11):202170077. doi: 10.1002/aisy.202170077

[14] Liu Y, Zhao G, Li G, He W, Zhong C. Analytical robust design optimization based on a hybrid surrogate model by combining polynomial chaos expansion and Gaussian kernel. Struct Multidisc Optim. 2022;65(11):335. doi: 10.1007/s00158-022-03400-z

[15] de Paula Garcia R, de Lima BSLP, de Castro Lemonge AC, Jacob BP. An enhanced surrogate-assisted differential evolution for constrained optimization problems. Soft Comput. 2023;27(10):6391–414. doi: 10.1007/s00500-023-07845-2

[16] Kaveh M, Mesgari MS. Application of meta-heuristic algorithms for training neural networks and deep learning architectures: a comprehensive review. Neural Process Lett. 2022:1–104. doi: 10.1007/s11063-022-11055-6 36339645 PMC9628382

[17] Al R, Behera CR, Gernaey KV, Sin G. Stochastic simulation-based superstructure optimization framework for process synthesis and design under uncertainty. Comput Chem Eng. 2020;143:107118. doi: 10.1016/j.compchemeng.2020.107118

[18] Marvi-Mashhadi M, Lopes CS, LLorca J. High fidelity simulation of the mechanical behavior of closed-cell polyurethane foams. J Mech Phys Solids. 2020;135:103814. doi: 10.1016/j.jmps.2019.103814

[19] Wang L, Chen X, Kang S, Deng X, Jin R. Meta-modeling of high-fidelity FEA simulation for efficient product and process design in additive manufacturing. Addit Manuf. 2020;35:101211. doi: 10.1016/j.addma.2020.101211

[20] Diao K, Sun X, Lei G, Guo Y, Zhu J. Multimode optimization of switched reluctance machines in hybrid electric vehicles. IEEE Trans Energy Convers. 2021;36(3):2217–26. doi: 10.1109/tec.2020.3046721

[21] Jin Z, Sun X, Cai Y, Zhu J, Lei G, Guo Y. Comprehensive sensitivity and cross-factor variance analysis-based multi-objective design optimization of a 3-DOF hybrid magnetic bearing. IEEE Trans Magn. 2021;57(2):1–4. doi: 10.1109/tmag.2020.3005446

[22] Ma C, Qu L. Multiobjective optimization of switched reluctance motors based on design of experiments and particle swarm optimization. IEEE Trans Energy Convers. 2015;30(3):1144–53. doi: 10.1109/tec.2015.2411677

[23] Dos Santos Neto PJ, dos Santos Barros TA, de Paula MV, de Souza RR, Ruppert Filho E. Design of computational experiment for performance optimization of a switched reluctance generator in wind systems. IEEE Trans Energy Convers. 2017;33(1):406–19. doi: 10.1109/tec.2017.2755590

[24] Cai J, Deng ZQ, Qi RY, Liu ZY, Cai YH. A novel BVC-RBF neural network based system simulation model for switched reluctance motor. IEEE Trans Magn. 2011;47(4):830–8. doi: 10.1109/tmag.2011.2105273

[25] Sahraoui H, Zeroug H, Toliyat HA. Switched reluctance motor design using neural-network method with static finite-element simulation. IEEE Trans Magn. 2007;43(12):4089–95. doi: 10.1109/tmag.2007.907990

[26] Koziel S, Leifsson L. Surrogate-based aerodynamic shape optimization by variable-resolution models. AIAA J. 2013;51(1):94–106. doi: 10.2514/1.j051583

[27] Owoyele O, Pal P. A novel machine learning-based optimization algorithm (ActivO) for accelerating simulation-driven engine design. Appl Energy. 2021;285:116455. doi: 10.1016/j.apenergy.2021.116455

[28] Thakur A, Chakraborty S. A deep learning based surrogate model for stochastic simulators. Probabilistic Eng Mech. 2022;68:103248. doi: 10.1016/j.probengmech.2022.103248

[29] Alizadeh R, Allen JK, Mistree F. Managing computational complexity using surrogate models: a critical review. Res Eng Design. 2020;31(3):275–98. doi: 10.1007/s00163-020-00336-7

[30] Nyshadham C, Rupp M, Bekker B, Shapeev AV, Mueller T, Rosenbrock CW, et al. Machine-learned multi-system surrogate models for materials prediction. npj Comput Mater. 2019;5(1). doi: 10.1038/s41524-019-0189-9

[31] Williams B, Cremaschi S. Novel tool for selecting surrogate modeling techniques for surface approximation. Comput Aided Chem Eng. 2021:451–6. doi: 10.1016/b978-0-323-88506-5.50071-1

[32] Davis SE, Cremaschi S, Eden MR. Efficient surrogate model development: impact of sample size and underlying model dimensions. Comput Aided Chem Eng. 2018:979–84. doi: 10.1016/b978-0-444-64241-7.50158-0

[33] Queipo NV, Haftka RT, Shyy W, Goel T, Vaidyanathan R, Kevin Tucker P. Surrogate-based analysis and optimization. Prog Aerosp Sci. 2005;41(1):1–28. doi: 10.1016/j.paerosci.2005.02.001

[34] Enss GC, Kohler M, Krzyzak A, Platz R. Nonparametric quantile estimation based on surrogate models. IEEE Trans Inform Theory. 2016;62(10):5727–39. doi: 10.1109/tit.2016.2586080

[35] Bramerdorfer G, Zavoianu A-C, Silber S, Lughofer E, Amrhein W. Possibilities for speeding up the FE-based optimization of electrical machines—a case study. IEEE Trans Ind Appl. 2016;52(6):4668–77. doi: 10.1109/tia.2016.2587702

[36] Kim S, Ki S, Bang S, Han S, Seo J, Ahn C, et al. Optimizing energy-efficient jet impingement cooling using an artificial neural network (ANN) surrogate model for high heat flux semiconductors. Appl Therm Eng. 2024;239:122101. doi: 10.1016/j.applthermaleng.2023.122101

[37] Ferreira S, Gunay B, Wills A, Rizvi F. A neural network-based surrogate model to predict building features from heating and cooling load signatures. J Build Perform Simul. 2024;17(5):631–54. doi: 10.1080/19401493.2024.2375304

[38] Baisthakur S, Fitzgerald B. Physics-informed neural network surrogate model for bypassing blade element momentum theory in wind turbine aerodynamic load estimation. Renew Energy. 2024;224:120122. doi: 10.1016/j.renene.2024.120122

[39] Li Z, Yu J, Wang C, Bello IT, Yu N, Chen X, et al. Multi-objective optimization of protonic ceramic electrolysis cells based on a deep neural network surrogate model. Appl Energy. 2024;365:123236. doi: 10.1016/j.apenergy.2024.123236

[40] Kolda TG, Lewis RM, Torczon V. Optimization by direct search: new perspectives on some classical and modern methods. SIAM Rev. 2003;45(3):385–482. doi: 10.1137/s003614450242889

[41] Haupt R. Comparison between genetic and gradient-based optimization algorithms for solving electromagnetics problems. IEEE Trans Magn. 1995;31(3):1932–5. doi: 10.1109/20.376418

[42] Meza JC. Steepest descent. WIREs Comput Stat. 2010;2(6):719–22. doi: 10.1002/wics.117

[43] Nocedal J, Wright SJ. Conjugate gradient methods. Num Optim. 2006:101–34.

[44] , Jr. JE, Moré JJ. Quasi-Newton methods, motivation and theory. SIAM Rev. 1977;19(1):46–89. doi: 10.1137/1019005

[45] Nocedal J, Wright SJ. Numerical optimization. New York, NY: Springer; 1999.

[46] Fletcher R. Practical methods of optimization. Wiley; 2013.

[47] Kraft D. A software package for sequential quadratic programming. Forschungsbericht Deutsche Forschungs- und Versuchsanstalt für Luft- und Raumfahrt. WorldCat; 1988.

[48] Han SP. A globally convergent method for nonlinear programming. J Optim Theory Appl. 1977;22(3):297–309. doi: 10.1007/bf00932858

[49] Powell MJD. The convergence of variable metric methods for nonlinearly constrained optimization calculations. Nonlinear Program. 1978;3:27–63. doi: 10.1016/b978-0-12-468660-1.50007-4

[50] Meunier L, Rakotoarison H, Wong PK, Roziere B, Rapin J, Teytaud O, et al. Black-box optimization revisited: improving algorithm selection wizards through massive benchmarking. IEEE Trans Evol Computat. 2022;26(3):490–500. doi: 10.1109/tevc.2021.3108185

[51] Rapin J, Teytaud O. Nevergrad - a gradient-free optimization platform. GitHub Repository. 2018.

[52] Bennet P, Langevin D, Essoual C, Khaireh-Walieh A, Teytaud O, Wiecha P, Moreau A. An illustrated tutorial on global optimization in nanophotonics. arXiv, preprint, arXiv:2309.09760. 2023.

[53] Kokash N. An introduction to heuristic algorithms. Department of Informatics and Telecommunications. 2005:1–8.

[54] Holland JH. Genetic algorithms. Sci Am. 1992;267(1):66–72. doi: 10.1038/scientificamerican0792-661411454

[55] Kennedy J, Eberhart R. Particle swarm optimization. In: Proceedings of ICNN’95 - International Conference on Neural Networks, vol 4, 1995, pp. 1942–8. doi: 10.1109/icnn.1995.488968

[56] Li F, Yue Q, Liu Y, Ouyang H, Gu F. A fast density peak clustering based particle swarm optimizer for dynamic optimization. Expert Syst Appl. 2024;236:121254. doi: 10.1016/j.eswa.2023.121254

[57] Bertsimas D, Tsitsiklis J. Simulated annealing. Stat Sci. 1993;8(1):10–15.

[58] Chen T, Guestrin C. XGBoost: A scalable tree boosting system. In: Proceedings of the 22nd ACM SIGKDD international conference on knowledge discovery and data mining. 2016:785–94.

[59] Friedman JH. Greedy function approximation: a gradient boosting machine. Ann Statist. 2001:1189–232. doi: 10.1214/aos/1013203451

[60] Kingma DP, Ba J. Adam: a method for stochastic optimization. arXiv, preprint, arXiv:1412.6980. 2014.

[61] Ke G, Meng Q, Finley T, Wang T, Chen W, Ma W, et al. LightGBM: a highly efficient gradient boosting decision tree. In: Advances in Neural Information Processing Systems 30, 2017.

[62] Rosenbrock HH. An automatic method for finding the greatest or least value of a function. Comput J. 1960;3(3):175–84. doi: 10.1093/comjnl/3.3.175

[63] Goodman J, Weare J. Ensemble samplers with affine invariance. CAMCoS. 2010;5(1):65–80. doi: 10.2140/camcos.2010.5.65

[64] Laguna M, Martí R. Experimental testing of advanced scatter search designs for global optimization of multimodal functions. J Glob Optim. 2005;33(2):235–55. doi: 10.1007/s10898-004-1936-z

